# Cascade Pumping Overcomes Hydraulic Resistance and Moderates Shear Conditions for Slow Gelatin Fiber Shaping in Narrow Tubes

**DOI:** 10.1016/j.isci.2020.101228

**Published:** 2020-06-02

**Authors:** Yuanxiong Cao, Haoran Zhao, Zhiwei Hu, Shaohua Ma

**Affiliations:** 1 Tsinghua-Berkeley Shenzhen Institute (TBSI), Tsinghua University, Shenzhen, 518055, China; 2 Tsinghua Shenzhen International Graduate School (SIGS), Tsinghua University, Shenzhen, 518055, China

**Keywords:** Materials in Biotechnology, Materials Processing, Biomaterials

## Abstract

In microextrusion-based 3D bioprinting, shaping gel fibers online, i.e., in narrow tubes, benefits the structural maintenance after extrusion, but it is challenging for materials possessing slow sol-gel transition dynamics. Gelatin, for example, transforms into thermostable fibers via transglutaminase (TG) reaction in as much as 10 min. It causes dramatic flow resistance accumulation and shear stress increase in fluids moving along narrow tubes, resulting in channel clogging and cell detriments. In this study, we overcome the limitations by adopting cascade pumping and performing in a single peristaltic pump that comprises multi-channel pumping units. The pressure and shear stress reduction by over 1-fold are verified by finite element simulation; continuous gelatin fiber production and patterning in a substrate-free manner are achieved via slow online enzymatic cross-linking. The online fiber shaping can be scaled up by numbering up the pumping units and provides another paradigm for biomanufacturing.

## Highlights

• Cascade pumping overcomes hydraulic resistance and reduces shear stress significantly
• Cascade pumping suits online gelatin fiber shaping via slow enzymatic cross-linking
• Online fiber shaping enables substrate-free 3D printing
• Gelatin fibers are stronger when gelled via thermal and enzymatic dual cross-linking

## Graphical Abstract

**Figure:**
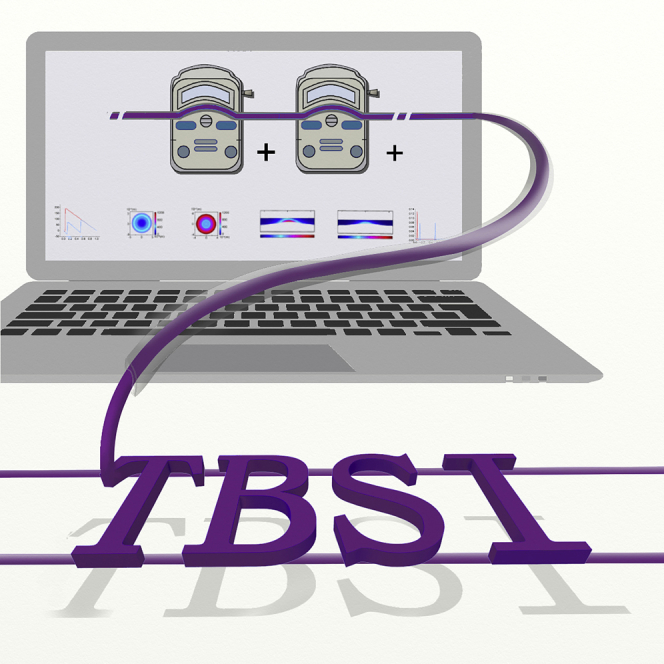

## Introduction

Additive manufacturing, such as microextrusion-based 3D bioprinting (Murphy and Atala, 2014, Shang et al., 2019), has developed into a prevalent technology in tissue engineering (Yu et al., 2018a, Yu et al., 2018b; Woodfield et al., 2004), regenerative medicine (Malda et al., 2013, Yu et al., 2018a, Yu et al., 2018b), and organ-on-a-chip-based precision medicine (Yi et al., 2019, Lee and Cho, 2016). During printing, the extruded fibers are usually shaped after being extruded from the nozzle. Microextrusion of online-shaped fibers, i.e., shaped in the flowing channel, overcomes the post-extrusion deformation and eliminates the dependence on printing supports (Bhattacharjee et al., 2015, Hinton et al., 2015, Ouyang et al., 2017). Gelatin is an extensively used biomaterial, shares comparable chemical compositions as collagen (Zeugolis et al., 2008, Elzoghby et al., 2012), and is endowed with appropriate biodegradability (Li et al., 2014) and mechano-regulation properties. It is also engineerable and low cost (Huang et al., 2009) and, therefore, becomes an ideal selection for scaled-up manufacturing of scaffolds for *in vitro* cell growth and implantation.

However, additional cross-linking shall be introduced to strengthen the thermal stability of gelatin at physiological conditions. Photo-cross-linking is effective to generate shaped fibers with enhanced thermostability during micro-extrusion (Ouyang et al., 2017), but it is potentially harmful to encapsulated cells (Noshadi et al., 2017) and requires chemical functionalization of native gelatin with reactive chemical groups, e.g., methacrylate (Nichol et al., 2010). Chemical cross-linking using glutaraldehyde is fast and effective to improve thermostability (Migneault et al., 2004), but the cross-linking agent is cytotoxic (Mi et al., 2006). Transglutaminase (TG)-catalyzed cross-linking is easy to operate, by simply mixing the enzyme with the native gelatin (Kolesky et al., 2016, Da Silva et al., 2014). Cross-linking proceeds with the inter-chain bonding (Cordella-Miele et al., 1990) at mild conditions, which almost has no damage to cells. However, it is challenging to shape gelatin via online cross-linking due to the slow reaction dynamics of gelatin and TG (Sakamoto et al., 1994). The incubation is required to be prolonged, for example, to nearly 10 min to accomplish the sol-gel transition. Long incubation of moving fluids in microchannel accumulates the hydraulic resistance (Oh et al., 2012), which often leads to the occurrence of channel clogging.

Another concern that cannot be surpassed in microextrusion is the shear stress in the flow volume, as a natural consequence of viscous fluid flows with a velocity gradient with their neighboring layers and friction with the channel boundaries (Yang et al., 2018). Newtonian fluids flow in microchannels are known to be confined by the channel boundaries, where the fluid layer immediately adjacent to the channel walls is not moving. The fluid in the channel core possesses the maximum velocity within a cross-section. Therefore, from the core to the boundary, a velocity gradient forms, generating local shear stress that correlates linearly with the velocity gradient and the fluid viscosity (Eskin, 2017). Shear stress, comprising in-plane shear and extensional strain components, can be taken advantage of to accelerate substance mixing (Zhao et al., 2015), but it is proven highly detrimental to encapsulated cells when exceeding certain levels (Brouzes et al., 2009). Therefore, reducing the shear stress levels in moving microfluids by rational design of fluid actuation has been under exploration and remains to be overcome in the academies of both microfluidics and 3D bioprinting (Saunders et al., 2008).

To tackle these problems, we designed a fluidic reaction system in a single peristaltic pump comprising multiple pumping channels. It was expected to overcome the hydraulic resistance accumulated when moving in a long tube, as the actuation powers were summed up from multiple or cascade pumping units. But the reduction on each pumping load, as well as the changes in the fluidic shear environment under different conditions, must be verified first. In this study, we compared the performance of single pumping and dual pumping, as a simple presentation of cascade pumping, on generating online-shaped gelatin fibers via the slow transglutaminase reaction. We simulated the flow and pumping conditions and verified the shear reduction by adopting the cascade pumping strategy. Afterward, dual pumping was proven advantageous against single pumping in generating gelatin fibers by avoiding the channel clogging and providing stable and directional gelatin actuation. Cell encapsulation was also a demonstrated success with a range of cell types. The shaped fibers were printed in both substrate-supporting and support-free manners, with the fibers shaped online at 25°C, via the simultaneous occurrence of cross-chain binding and peptide assembly, exhibiting higher tensile strength than fibers gelled at 37°C, via enzymatic cross-chain binding only, or fibers gelled at 25°C in the absence of TG, via peptide assembly only.

## Results

### Cascade Actuation for Online Synthesis of Gelatin Fibers

**Figure 1:**
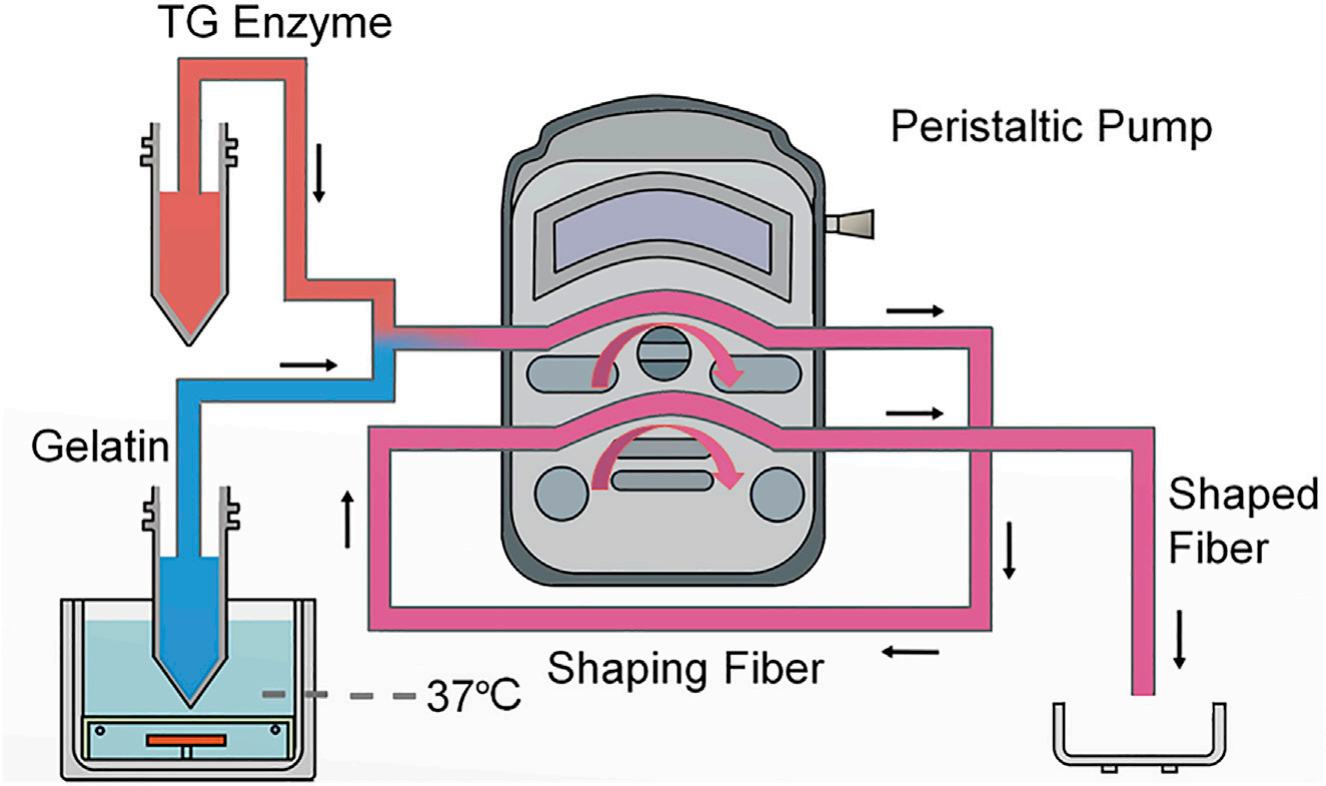
Sketch of Cascade Pumping for Online Gelatin Fiber Synthesis. The multi-channel peristaltic pump provides consecutive actuation that withdraws and infuses gelatin and TG solutions in the mixing and reaction tube and drives the directional locomotion of shaping gelatin fibers toward the outlet.

The cascade actuation for online gelatin fiber synthesis is sketched in Figure 1. The gelatin (6%, w/v) and transglutaminase (TG, 60%, w/v) solutions stored separately in two 50-mL centrifuge tubes were connected to two ports of a plastic T-shape connector via two flexible silicone tubes (inner diameter [ID] = 1.6 mm). Gelatin was incubated at 37°C to prevent gelation. A long flexible silicone tube (ID = 1.6 mm) was connected to the third port of the connector and then fitted to a channel inside the pump casing (Transparent methods). The pump withdrew and infused the homogenized solution in a synchronized manner as the fluids were incompressible. The tube was then fitted to another channel in the pump casing, to consecutively withdraw and infuse the fluid. The consecutive pumping effectively overcame the hydraulic resistance accumulated in a long tube, avoided channel clogging by the gelling fluid. The sol-gel transition resulted from the enzymatic reaction on gelatin catalyzed by TG and the peptide assembly on gelatin itself at 25°C. Cascade pumping neutralized or reduced the stress accumulated in each segment rather than passing it down to the next segment, which, therefore, reduced the overall stress accumulation in the entire flow system.

The number of cascade pumping unit is unlimited, thus allowing the mixing and reaction tube to be extended as long as demanded. Therefore, it suits slow reactions to occur in moving fluids in a narrow tube. For this study, the TG-catalyzed gelatin cross-linking took over 10 min to accomplish at either 25°C or 37°C, characterized by solid fiber formation at the tube outlet. We adopted the design of two consecutive peristaltic pumpings to provide sufficient actuation to overcome the flow resistance accompanying the fiber shaping in tubes.

A single peristaltic pump comprising 16 channels can, in principle, provide as many as 16 consecutive repeats of fluid withdrawal and infusion, by which means actuation for flow reactions taking over a few hours could be accomplished in a narrow tube. The entire system relies on only one single peristaltic pump. The production can be scaled up by numbering up the pumpings. Apart from these apparent advantages, we also speculated that the strategy of segmented actuation benefits cell encapsulation, by providing an equivalent sum of actuation powers but with significantly reduced shear magnitudes that are proven among the most detrimental factors to encapsulated cells in biomanufacturing.

### Significantly Reduced Shear Magnitudes in Cascade Pumping

**Figure 2:**
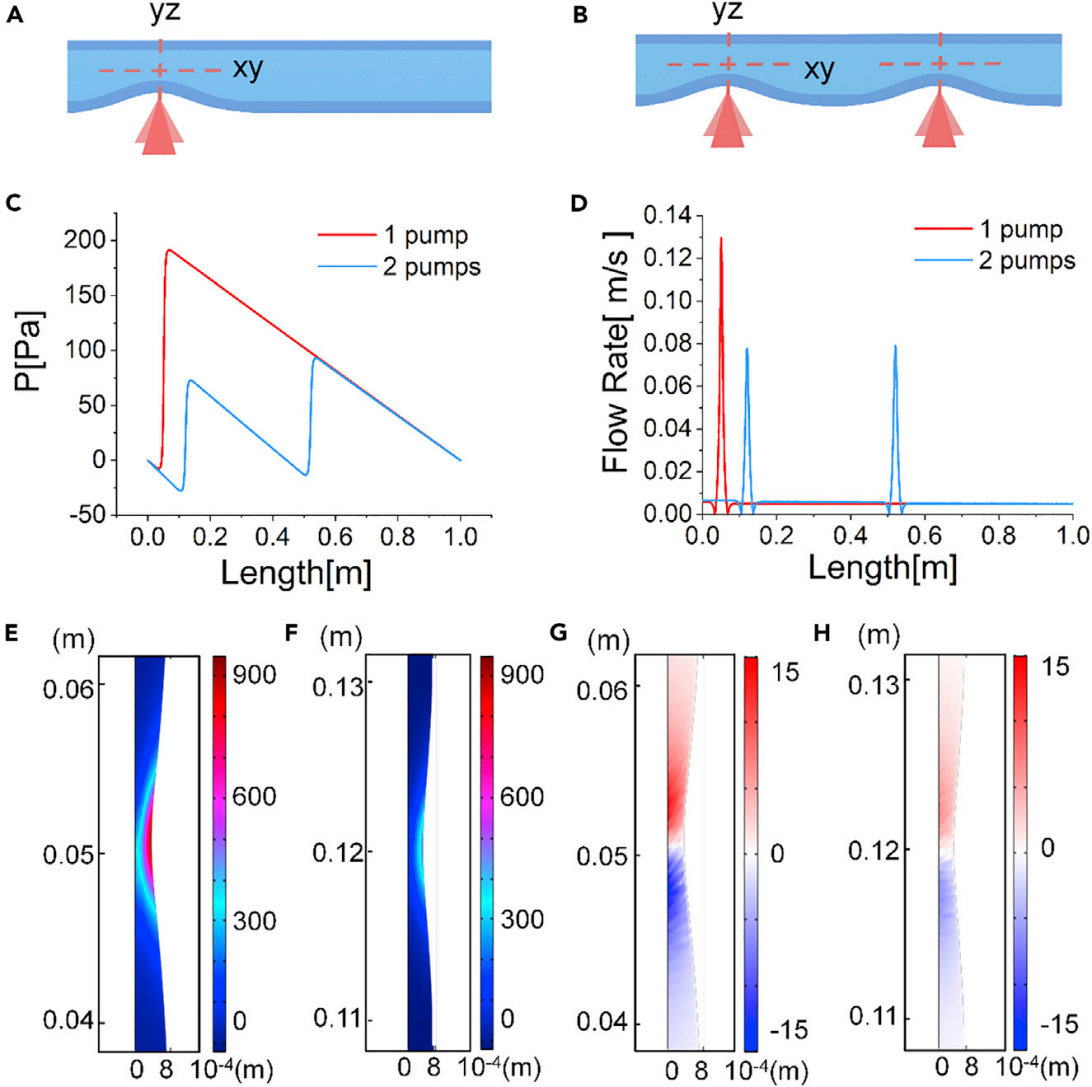
Flow and Shear Rate Comparison between Single and Dual Pumping (A and B) Sketch of the peristaltic pumping and investigated planes in (A) single pumping and (B) dual pumping. (C) Pressure distribution of the tubular fluid at *y* = r/2 along the tube axis. *r* is the inner radius of the tube. (D) Flow rate distribution at *y* = r/2 along the tube axis. (E and F) Contours of in-plane shear rate of (E) single pumping and (F) dual pumping at the (first) pumping position. (G and H) Contours of extensional strain of (G) single pumping and (H) dual pumping at the (first) pumping position.

Shear stress is an important factor affecting cell viability. We used finite element simulation to investigate the shear magnitudes in single and cascade pumping, which was represented by dual pumping in this study (Figures 2A and 2B). Throughout the simulation, the tube length was set as 1 m (Table S1). The pumping position was set at 0.1 m for the single pumping, and 0.1, 0.5 m, respectively, for the dual pumping, all in reference to the tube inlet. The length of tube deformation was set constant as 4 cm for both pumpings. The hydraulic pressure along the tube axis was significantly higher in the first half of the tube, approximately from 0 to 0.5 m, for the single pumping than for the dual pumping. The pressure in the single pumping condition reached its maximum value at approaching the pumping location and formed a linear decrease profile to the outlet. On the contrary, the maximum pressure in the dual pumping condition, which was even below half the magnitude as in the former condition, shifted for approximately 0.1 m toward the outlet and formed a linear decrease profile till approaching the secondary pumping, where a similar profile formed as its precedent. The pressures in both conditions remained nearly identical in the second half of the tube, i.e., from 0.5 to 1 m, when the pressures in both conditions dropped linearly from a certain value to 0 (Figure 2C).

As we set the power supply from both conditions to be identical, the load afforded by each pumping unit would be different; the single pumping required a higher power supply that equaled the sum supply from the dual pumping, reflected as a larger degree of tube deformation for the single pumping than for the dual pumping. Therefore, at the pumping position, the peak flow rate of the single pumping was ∼1-fold higher than that of the dual pumping. Apart from the peak values, the flow rates in constant flow in both conditions remained the same and 1–2 orders of magnitude lower than the peak ones (Figure 2D).

To evaluate the shear environment for cell encapsulation applications, the in-plane and extensional shear strain rates were estimated from the computed velocity fields. Hydrodynamic stresses act in all three dimensions and hence accurate estimation of the shear rate magnitude requires both elongational and shear strain terms to be accounted for (Kaliviotis et al., 2011):

(Equation 1) $$\;\left|\dot{\gamma }\right|={\left[2{\left(\frac{\partial u}{\partial x}\right)}^{2}+2{\left(\frac{\partial v}{\partial y}\right)}^{2}+2{\left(\frac{\partial w}{\partial z}\right)}^{2}+{\left(\frac{\partial v}{\partial x}+\frac{\partial u}{\partial y}\right)}^{2}+{\left(\frac{\partial w}{\partial y}+\frac{\partial v}{\partial z}\right)}^{2}+{\left(\frac{\partial u}{\partial z}+\frac{\partial w}{\partial x}\right)}^{2}\right]}^{1/2}\;$$

This total shear rate was calculated after all nine velocity gradients were obtained. In the fluid pumping condition, because the tube was axisymmetric, the *z* velocity component, *w*, had the same order of magnitude as the *y* velocity component, *v*, and both components were about 2 orders of magnitude smaller than the *x* velocity component, *u*. Therefore, the vector differences between *w* and *v* were ignored, and both employed the value from *v*. In the *xy* plane, there are two shear components, the in-plane shear and the extensional strain, the rates of which were calculated from $\varepsilon =\partial \bar{u}/\partial y+\partial \bar{v}/\partial x$ and $\text{η}=\partial \bar{u}/\partial x+\partial \bar{v}/\partial y$, respectively (Ma et al., 2015).

Both the in-plane shear rate (Figures 2E and 2F) and the extensional strain rate (Figures 2G and 2H) in the single pumping condition were much higher than in the dual pumping condition at the proximal of the pumping positions. The maximum values of the in-plane shear and the extensional strain rates were approximately 885 and 14 s^−1^ for the single pumping (Figures 2E and 2G), 435 and 6.75 s^−1^ at the first pumping position (Figures 2F and 2H), and 458 and 7.02 s^−1^ (Figure S1) at the second pumping position for the dual pumping condition. Both shear components in the dual pumping were substantially reduced than their counterparts in the single pumping. Therefore, cascade pumping might be concluded to moderate the shear environment and benefit gel fibers shaping in narrow tubes when cells are encapsulated.

**Figure 3:**
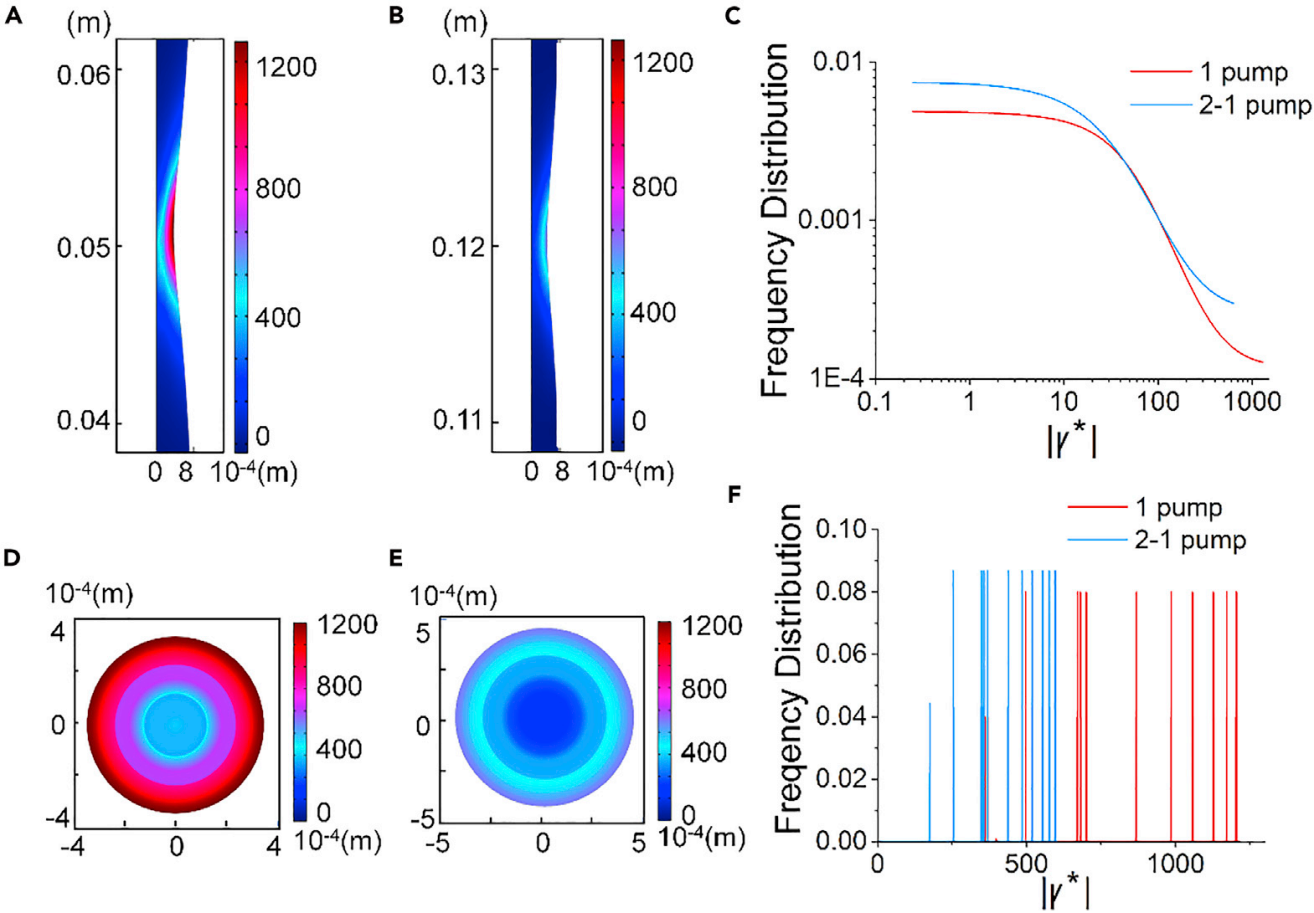
Contours and Shear Rate Frequency Distribution on the *xy* and *yz* Planes (A–C) Contours and shear rate frequency distribution on the *xy* plane with (A) single pumping, (B) dual pumping at its first pumping position, and (C) the frequency distribution of shear magnitudes at the two conditions. (D–F) Contours and shear rate frequency distribution on the *yz* plane with (D) single pumping, (E) dual pumping at its first pumping position, and (F) the frequency distribution of shear magnitudes at the two conditions.

To further elucidate the advantages of cascade (*or* dual) pumping for cell encapsulation, the shear rate magnitudes $\left|\gamma^{*}\right|$ at the *xy* and *yz* planes of the single and dual pumping were calculated using Equation (1) (Figures 3A, 3B, and S2). $\left|\gamma^{*}\right|$ reached 1,285 s^−1^ in the *xy* plane for single pumping (Figure 3A), whereas the magnitudes topped at 624 and 643 s^−1^ for the two consecutive pumping positions (Figures 3B and S2A) in dual pumping. The latter substantially reduced the shear level by splitting the pumping load into two pumpings. The frequency of shear rates below 100 s^−1^ showed no obvious difference between the single and dual pumping conditions. At higher rates, e.g., above 100 s^−1^ but smaller than 600 s^−1^, the dual pumping condition gained higher frequency than the single pumping, but it topped at near 600 s^−1^; contrarily, the single pumping generated higher shear rates that extended to nearly 1,300 s^−1^. As the higher shear rate magnitude is known to be substantially more detrimental to cells than the lower ones, the lower frequency of shear rates in the medium-high region, i.e., from 100 to 600 s^−1^, is not compensating the harm caused by even higher rates (Figures 3C and S2B).

The shear rate magnitudes $\left|\gamma^{*}\right|$ at the *yz* plane are shown in Figures 3D, 3E, and S2C. The maximum shear rate at the *yz* plane for single pumping was 1,204 s^−1^. The rates only reached 597 s^−1^ (at the first pumping position) and 622 s^−1^ (at the second pumping position) for dual pumping. The frequency distribution at the single and dual pumping conditions on the *yz* plane was profoundly different. The single pumping condition dominated the frequency distribution of shear rates higher than 600 s^−1^, whereas the dual pumping condition only comprised shear rates lower than 600 s^−1^ (Figure 3F). There was no obvious difference between the first and second pumping positions (Figure S2D) for the dual pumping.

The shear environment is weakened when using dual pumping to shear the fluid actuation load that would have been afforded by single pumping. We could also derive that cascade pumping consisting of consecutive pumping operations could perform tough tasks, e.g., a longer tube carrying reacting and solidifying fluids, because cascade pumping could provide more power than single pumping when each hard piece applied the same amount of force on the driving fluid (Figure S3). It could also ease a task by splitting the load afforded by a single pumping and, meanwhile, moderate the shear environment for the substances being pumped.

### Shear Rate Comparison by Dual Pumping at Different Distances

**Figure 4:**
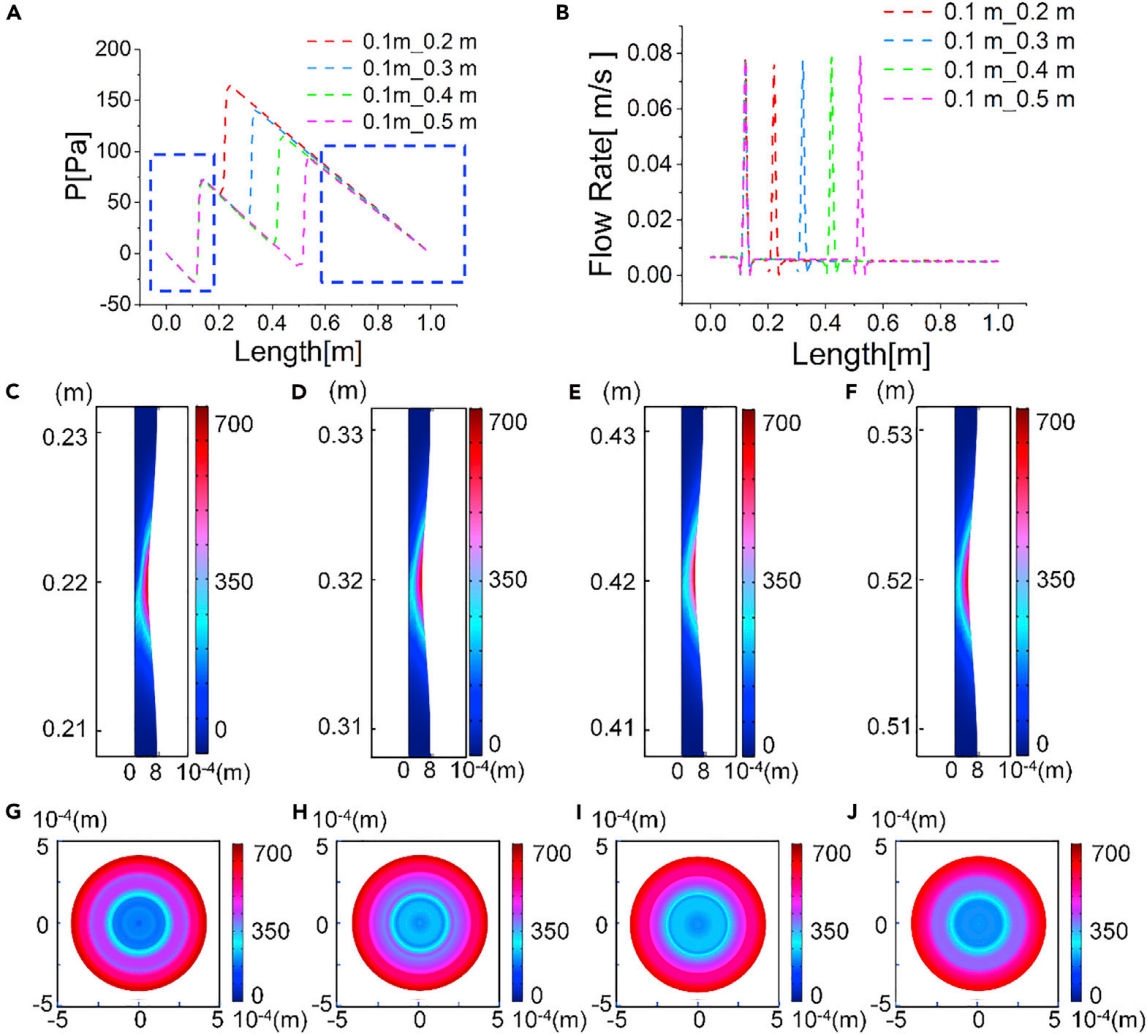
Shear Rate Comparison of Dual Pumping at Different Distances The first pumping position was fixed at 0.1 m; the second pumping position was fixed at 0.2, 0.3, 0.4, 0.5 m, respectively. All distances were referred to the tube inlet. (A) Pressure distribution of the tubular fluid core, i.e., at *y* = r/2, along the tube axis, when pumped at different distances. (B) Flow rate distribution of the fluid core along the tube axis. (C–F) Contours of shear rates $\left|\gamma^{*}\right|$ on the *xy* plane at the secondary pumping position of different distances: (C) 0.2, (D) 0.3, (E) 0.4, and (F) 0.5 m. (G–J) Contours of shear rates on the *yz* plane at the secondary pumping position of different distances: (G) 0.2, (H) 0.3, (I) 0.4, and (J) 0.5 m.

To investigate whether the positioning of cascade pumping affects the shear environment, we computed the flow rates and shear magnitudes at the proximal of the pumping positions at different distances. The first pumping position for dual pumping was fixed at 0.1 m; the secondary pumping was fixed at 0.2, 0.3, 0.4, 0.5 m, respectively. The fluid pressure distribution from the inlet to the first pumping and from the secondary pumping to the outlet remained the same (Figure 4A). The pressure difference was minimized with the increase of the secondary pumping distance. In other words, the pressure at the secondary pumping position was maximum when it was close to the first pumping (Figure 4A). The peak values and the distribution of flow rates at all the pumping positions and distances were nearly identical (Figure 4B). The shear rate magnitudes $\left|\gamma^{*}\right|$ on the *xy* and *yz* planes were calculated using Equation (1). The shear rates on the *xy* plane displayed similar topology, and their maximum values at the secondary pumping position were 670, 658, 648, and 643 s^−1^, when the distances were fixed at 0.2, 0.3, 0.4, 0.5 m, sequentially (Figures 4C–4F). The maximum shear rates on the *yz* plane at the secondary pumping position were 646, 638, 626, and 622 s^−1^, respectively. Their frequency distributions showed no obvious difference (Figures S4A and S4B). The shear rate magnitude $\left|\gamma^{*}\right|$ at the fixed first pumping position was computed. At all distances of the secondary pumping, the shear environment remained unchanged (Figure S5).

### Characterization of the Shaped Gelatin Fibers

**Figure 5:**
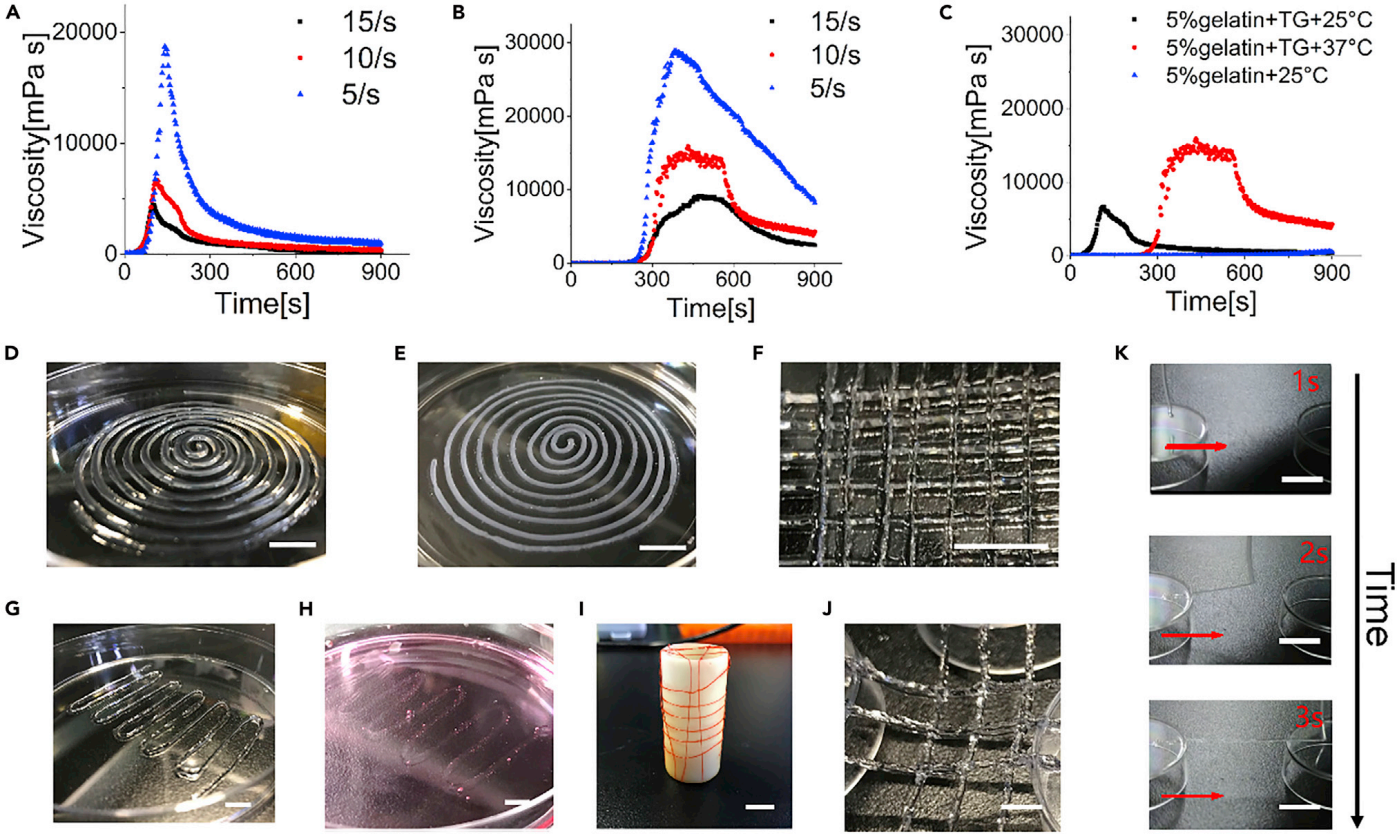
Characterization of the Gelatin/TG Mixed Solution and the Shaped Gelatin Fibers (A and B) Time-dependent viscosity of 5% (w/v) gelatin solution, containing the saturated TG solution, at the shear rates of 5, 10, 15 s^−1^ at (A) 25°C and (B) 37°C. (C) Time-dependent viscosity of 5% (w/v) gelatin/TG solutions at 25°C and 30°C and the 5% (w/v) gelatin-only solution at 25°C, sheared at the rate of 10 s^−1^. The measurement started at the time point of mixing gelatin and TG. (D) Spiral patterning of a single gelatin fiber (1 m long). (E) The fibers were immersed in PBS buffer for 24 h. (F) The fibers were written in cross-shapes. (G) The fibers were written in a zigzag pattern. (H) The fibers were immersed in Dulbecco's modified Eagle's medium (DMEM) for 24 h (I and J) The fibers were patterned in orthogonal orders (I) with the ends anchored on a cylinder and (J) to plastic plates. (K) The fibers were patterned without substrate support. Scale bars in all panels: 1 cm.

The gelatin/TG solution underwent sol-gel transition in the reaction tube and shaped into fibers. Figure 5A shows that the viscosity of the gelatin/TG solution (25°C) increased first and then decreased till reaching a plateau with time at constant shear rates. The viscosity decreased with increased shear rates, exhibiting shear-thinning properties. When increasing the gelatin/TG solution to 37°C, the trend was exactly the same, including the appearance of the shear-thinning properties (Figure 5B). But when shearing the gelatin solution (25°C) at 10 s^−1^, it remained at extremely low viscosity for 9 min (Figure 5C) and failed to reach a constant level within 1 h constant shearing (Figure S6), owing to the slow dynamics of gelatin gelation via peptide assembly at 25°C. While comparing gelatin/TG solution at 25°C and 37°C at the shear rate of 10 s^−1^, the viscosity of gelatin/TG solution (25°C) was lower than gelatin/TG solution (37°C) (Figure 5C), which was attributed to the accelerated TG cross-linking reaction at elevated temperatures. It also suggested that flowing gelatin/TG solution at 25°C overcame less hydraulic resistance and experienced less shear than flowing at 37°C.

The gelatin solution then was cross-linked by the gradual enzymatic reaction in the shaping tubing and formed a fiber before approaching the nozzle and being extruded. The fibers can be arranged in designed patterns by simply “writing” in the supporting media or in substrate-free manners, such as spiral (Figure 5D), cross-shaped (Figure 5F), and zigzag (Figure 5G) patterns in a Petri dish. The fibers and patterns remained highly stable while immersed in PBS (Figure 5E) or DMEM (Figure 5H) for 24 h. The diameters of gelatin fiber were measured before and after 24 h in deionized water (DI water), PBS, and DMEM, respectively. Fibers in all conditions became narrowed by 38.46%, 22.83%, and 23.32%, in DI water, PBS, and DMEM, respectively, suggesting that the incubation condition and medium influence the fiber morphology (Figure S7). However, in all conditions, the fibrous shapes and organizations were maintained.

The fibers were also twined on a cylindrical support, with tailorable intervals and retained strains (Figure 5I), and orderly patterned with retained strains in substrate-free manners, with only the ends being anchored to solid supports (Figures 5J and 5K, and Video S1).

Video S1. Substrate Free Printing, Related to Figure 5

The online fiber shaping eliminates the dependence of printing slow-cross-linking soft bio-scaffolds on solid supports or granular medium (Ouyang et al., 2017).

### Materials and SEM Characterization of Gelatin/TG Gel

**Figure 6:**
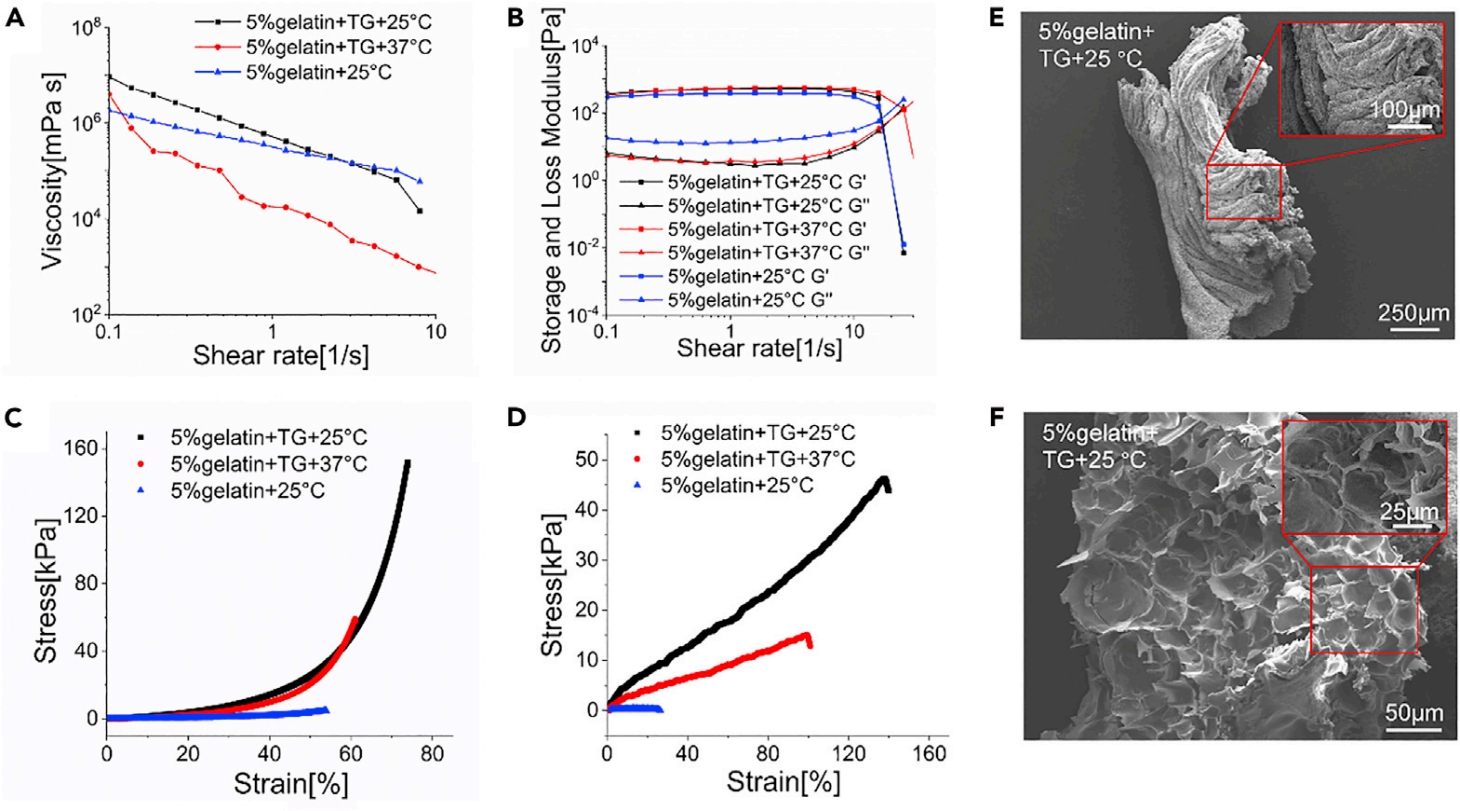
Rheology and Morphology (SEM) Characterization of Gelatin Gels under Different Conditions It compares the 5% (w/v) gelatin/TG gelled at 25°C and 37°C, respectively, and the 5% (w/v) gelatin only gelled at 25°C (A) The apparent viscosity of the three gels with increased shear rates. (B) The apparent storage moduli (G′) and loss moduli (G″) of the three gels with increased shear rates. (C) The compressive stress-strain curves of the three gels. (D) The tensile stress-strain curves of the three gels. (E and F) SEM images of a 5% (w/v) gelatin/TG fiber gelled at 25°C and immersed in DMEM medium for 1 week, imaged from the (E) longitudinal and (F) transverse directions. Gel shapes: (A, B, C) molded bulk gels; (D) gel fibers.

After the three groups of gelatin solutions transformed into hydrogels, it was found that these all showed shear thinning properties while increasing the shear rate from 0.1 to 10 s^−1^ (Figure 6A). For storage and loss modulus of these three kinds of hydrogel, the cross points of G′ (the storage modulus) and G″ (the loss modulus) were constant while increasing the shear rate from 0.1 to 15 s^−1^ (Figure 6B).

The compressive modulus of 5% (w/v) gelatin/TG hydrogel (25°C) reached 160 kPa and nearly 80% strain before failure, whereas the 5% gelatin/TG hydrogel (37°C) failed at 60 kPa and 60% strain (Figure 6C). The 5% gelatin gel (25°C) showed the lowest compressive modulus (10 kPa) and failure strain (55%). For tensile tests, the strain of gelatin/TG fiber (25°C) reached 1.4 and the tensile module was nearly 50 kPa (Figure 6D). But on increasing the temperature from 25°C to 37°C, the strain decreased by 35.7% and the tensile modulus decreased by 70%. The 5% gelatin (25°C) fiber exhibited the lowest failure strain and tensile modulus. It was proven that the mechanical properties of 5% gelatin/TG hydrogel (25°C) was significantly improved than the 5% gelatin/TG hydrogel (37°C) and 5% gelatin hydrogel (25°C). It implies that the simultaneous physical cross-linking via peptide assembly and enzymatic cross-linking via cross-peptide covalent bonding formation enhanced the elasticity of gelatin fibers, compared with fibers gelled by either one of the cross-linking mechanisms.

A 5% gelatin/TG (25°C) fiber was immersed in DMEM for 1 week. Microfibers emerged and aligned along the fiber axis, as shown in the SEM images (Figures 6E and 6F). The pore size was slightly enlarged than the fresh fibers, i.e., non-immersed or incubated in DMEM (Figures 6F and S8A). The hydration incubation enlarging porosity was further verified by comparing the fresh and 24-h-incubated 5% gelatin/TG gel in PBS (Figures S8A and S8C). For 5% gelatin/TG (37°C) fibers, the pore sizes were significantly larger than the 5% gelatin/TG (25°C) fibers (Figures S8A and S8B). The enhanced elasticity and strength of the 5% gelatin/TG (25°C) might be attributed to the increased density and uniformity of smaller pores.

### Cell Encapsulation with High Viability Retention

**Figure 7:**
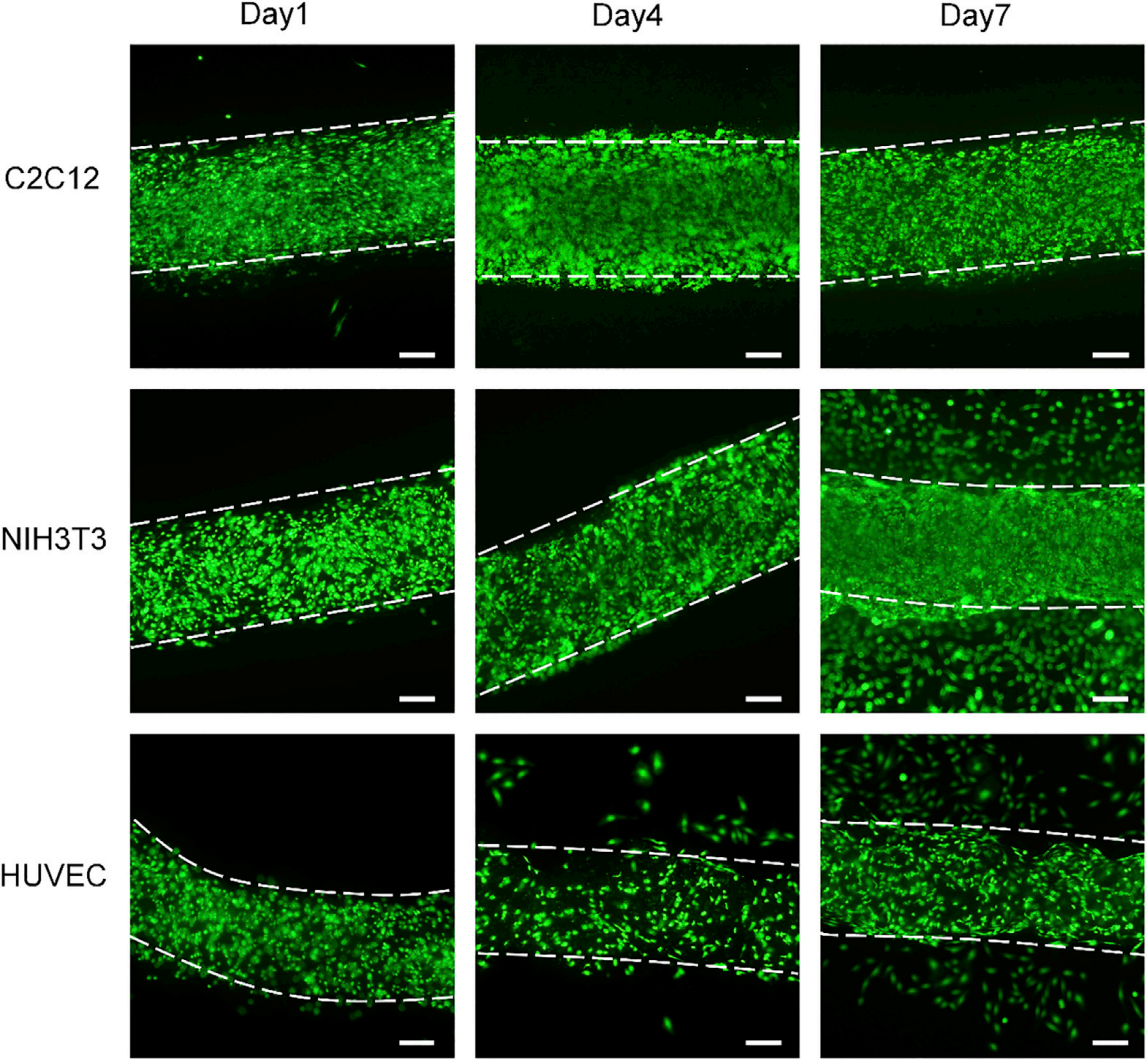
Cell Encapsulation with High Viability Retention Cultured gelatin/TG fibers with C2C12, NIH3T3, HUVEC cells for 1 day, 4 days, and 1 week formed at 25°C under dual pumping. Cells were stained with Calcein AM and PI, indicating the live and dead cells. The dead cells were rare and invisible (also shown in Figure S9). Scale bars in all panels: 200 μm.

Human umbilical vein endothelial cells (HUVECs), NIH3T3cells, and C2C12 skeletal muscle cells were used to evaluate the cell retention ability of gelatin fibers as anisotropic bio-scaffolds. The cells were first suspended in the gelatin solution at a seeding density of 3.0×10^6^ cells per mL before being loaded in the cartridge. The fiber synthesis followed the procedures as the synthesis of acellular fibers. Figure 7 shows the cells being encapsulated in the fibers at day 1, day 4, and day 7. The live (green) staining shows the high cell viability retention at both post-encapsulation and 1 week in culture. Figure S9 shows the live cells are dominant over the dead cells in counts. The results indicated that enzymatic cross-linking, together with the peptide assembly, endowed gelatin the thermal and structural stability and the supreme elasticity and cytocompatibility.

## Discussion

In this work, we present an online gel fiber shaping system specialized for slow cross-linking reaction rendered gelation. The production is not limited to gelatin. The multi-phase fluid flow is modulated by a single peristaltic pump comprising multi-channels. Our simulation study verifies that cascade pumping is efficient to moderate the environment in fluid volumes moving in confined narrow channels; experimental studies prove that cascade pumping overcomes the channel clogging issue resulting from the accumulation of hydrodynamic resistance, which is often seen in the long flow of viscous fluids or gelling fluids in biomanufacturing. One additional pumping unit reduces the maximum shear rate by over 1-fold as in the single pumping module, without sacrificing the online fiber production efficiency or increasing the setup complexity, as both single and cascade pumping are performed in a single peristaltic pump. The system overcomes the increased hydraulic resistance accompanying the prolonged incubation to compromise slow cross-linking reactions and avoids channel clogging. Eventually, it is proven that cells can be encapsulated in gelatin fibers with high viability retention. Moreover, the production modules could be numbered up, thus scaling up the production. For example, in a pump comprising 16 channels, 8 production modules can run in parallel. The online fiber shaping eliminates the dependence on solid substrates or granular medium to assist fiber shaping and stabilize fibrous patterns after extrusion. It broadens the application of 3D bioprinting, particularly, extending the microextrusion-based tissue engineering to *in situ* printing, where the recipient volume is mostly irregular and sometimes requires contactless assembly or residual strain after printing.

We did observe that, under single pumping, the flow was clogged, which was among the motivations for this innovation. After clogging, we managed to manually extrude the cell-laden fibers shaped in tubes and found that cells were recovered with lower viability and the fibers failed to retain their shapes (Figure S10).

Notably, the enzymatic cross-linking conducted at room temperature (e.g., 25°C) generates stronger gelatin fibers exhibiting larger failure stress and strain than fibers cross-linked at 37°C. At 25°C, the gelatin gelation proceeds via the peptide assembly modulated by thermal energy and the inter-peptide cross-linking induced by enzyme-catalyzed covalent bonds formation. The dual-cross-linking mechanism is proven advantageous against the sole cross-linking contributed by TG.

Consequently, the shear stress-reduced fiber shaping system as we designed can be widely used to print an extensive range of materials and cells. It increases the printing efficiency, reduces the chances of cell damage, and, meanwhile, is easily adaptable to other hardware modules, such as the locomotors as widely used in 3D bioprinting. Compared with the existing printing methods, the cascade pumping strategy shows advantages over the microextrusion printing and inkjet printing, in shear rate reduction, cell viability maintenance, compatibility with substrate-free printing, and reduced cost on the printer setup. Although the printing resolution is not as comparable with the laser-assisted printing, its cost is significantly lower and allows printing materials lack of light sensitivity (Table 1). The cascade pumping can be easily scaled up numbering up the printing channels. It, therefore, offers another paradigm for cell and tissue printing and promises to contribute to the development of precision and regenerative medicine.

**Table 1:** Comparison of *Cascade Pumping* and the Existing Printing Techniques

Type	Cascade Pumping Printing	Microextrusion Printing	Laser-Assisted Printing	Inkjet Printing
Shear rate reduction	+++	+	+++	++
Cell viability	+++	++	+++	+++
Substrate-free printing	+++	+	+++	+
Printing resolution	+	+	+++	+
Printing cost	+	++	+++	+

Low efficacy (+), moderate efficacy (++), high efficacy (+++).

### Limitations of the Study

Of course, there are limitations in the current system. First, it is difficult to precisely quantify the dual-phase mixing in the shaping tube. The volume ratio of fluids can be coarsely tailored to be withdrawn and infused into the mixing tube, by adjusting the hydrodynamic resistances of individual fluids. It can be done by choosing the sizes and lengths of the conducting tubes. However, this method is insufficient for precision fluid manipulation, especially when the fluids are distinctively different in viscosity. For example, gelatin is much more viscous than the TG solution, and its viscosity changes with temperature. It could be solved by using the combination of microfluidics pumps that infuse gelatin and TG solutions at precisely defined flow rates.

Second, it remains a challenge to produce fine fibers, e.g., smaller than 100 μm in diameter, as the hydraulic resistance increases following the power law of four with the decrease in the channel diameter. Third, peristaltic pump only works on tubes that are pliable to compression forces. But these materials are not guaranteed chemo-resistant and thermostable as polytetrafluoroethylene (PTFE) tubes. But PTFE tubes are not as pliable to external forces. A good material satisfying the pumping requirement but meanwhile possessing the thermal and chemical stability is highly demanded.

Despite the limitations, we believe this system has translational promises in the production of slow-reaction-rendered gel fibers for online (*or in situ*) and substrate-free tissue printing, especially for scale-up manufacturing of artificial organs.

### Resource Availability

#### Lead Contact

Further information and requests for resources and reagents should be directed to and will be fulfilled by the Lead Contact, Prof. Shaohua Ma (ma.shaohua@sz.tsinghua.edu.cn).

#### Materials Availability

This study did not generate new unique reagents.

#### Data and Code Availability

The simulation data that supports the findings of this study is available from the corresponding author upon reasonable request.

## Methods

All methods can be found in the accompanying Transparent Methods supplemental file.

## Supplemental Information

Document S1. Transparent Methods, Figures S1–S10, and Table S1

## References

[bib1] Bhattacharjee T., Zehnder S.M., Rowe K.G., Jain S., Nixon R.M., Sawyer W.G., Angelini T.E. (2015). Writing in the granular gel medium. Sci. Adv..

[bib2] Brouzes E., Medkova M., Savenelli N., Marran D., Twardowski M., Hutchison J.B., Rothberg J.M., Link D.R., Perrimon N., Samuels M.L. (2009). Droplet microfluidic technology for single-cell high-throughput screening. Proc. Natl. Acad. Sci. U S A.

[bib3] Cordella-Miele E., Miele L., Mukherjee A.B. (1990). A novel transglutaminase-mediated post-translational modification of phospholipase A2 dramatically increases its catalytic activity. J. Biol. Chem..

[bib4] Elzoghby A.O., Samy W.M., Elgindy N.A. (2012). Protein-based nanocarriers as promising drug and gene delivery systems. J. Control. Release.

[bib5] Eskin D. (2017). Modeling an effect of pipe diameter on turbulent drag reduction. Chem. Eng. Sci..

[bib6] Hinton T.J., Jallerat Q., Palchesko R.N., Park J.H., Grodzicki M.S., Shue H.J., Ramadan M.H., Hudson A.R., Feinberg A.W. (2015). Three-dimensional printing of complex biological structures by freeform reversible embedding of suspended hydrogels. Sci. Adv..

[bib7] Huang K.S., Lu K., Yeh C.S., Chung S.R., Lin C.H., Yang C.H., Dong Y.S. (2009). Microfluidic controlling monodisperse microdroplet for 5-fluorouracil loaded genipin-gelatin microcapsules. J. Control. Release.

[bib8] Kaliviotis E., Dusting J., Balabani S. (2011). Spatial variation of blood viscosity: modelling using shear fields measured by a μPIV based technique. Med. Eng. Phys..

[bib9] Kolesky D.B., Homan K.A., Skylar-Scott M.A., Lewis J.A. (2016). Three-dimensional bioprinting of thick vascularized tissues. Proc. Natl. Acad. Sci. U S A.

[bib10] Lee H., Cho D.W. (2016). One-step fabrication of an organ-on-a-chip with spatial heterogeneity using a 3D bioprinting technology. Lab. Chip.

[bib11] Li Y., Liu W., Liu F., Zeng Y., Zuo S., Feng S., Qi C., Wang B., Yan X., Khademhosseini A. (2014). Primed 3D injectable microniches enabling low-dosage cell therapy for critical limb ischemia. Proc. Natl. Acad. Sci. U S A.

[bib12] Ma S., Sherwood J.M., Huck W.T., Balabani S. (2015). The microenvironment of double emulsions in rectangular microchannels. Lab. Chip.

[bib13] Malda J., Visser J., Melchels F.P., Jüngst T., Hennink W.E., Dhert W.J., Hutmacher D.W. (2013). 25th anniversary article: engineering hydrogels for biofabrication. Adv. Mater..

[bib14] Mi F.L., Huang C.T., Liang H.F., Chen M.C., Chiu Y.L., Chen C.H., Sung H.W. (2006). Physicochemical, antimicrobial, and cytotoxic characteristics of a chitosan film cross-linked by a naturally occurring cross-linking agent, aglycone geniposidic acid. J. Agric. Food Chem..

[bib15] Migneault I., Dartiguenave C., Bertrand M.J., Waldron K.C. (2004). Glutaraldehyde: behavior in aqueous solution, reaction with proteins, and application to enzyme crosslinking. Biotechniques.

[bib16] Murphy S.V., Atala A. (2014). 3D bioprinting of tissues and organs. Nat. Biotechnol..

[bib17] Nichol J.W., Koshy S.T., Bae H., Hwang C.M., Yamanlar S., Khademhosseini A. (2010). Cell-laden microengineered gelatin methacrylate hydrogels. Biomaterials.

[bib18] Noshadi I., Hong S., Sullivan K.E., Sani E.S., Portillo-Lara R., Tamayol A., Shin S.R., Gao A.E., Stoppel W.L., Black L.D. (2017). In vitro and in vivo analysis of visible light crosslinkable gelatin methacryloyl (GelMA) hydrogels. Biomater. Sci..

[bib19] Oh K.W., Lee K., Ahn B., Furlani E.P. (2012). Design of pressure-driven microfluidic networks using electric circuit analogy. Lab. Chip.

[bib20] Ouyang L., Highley C.B., Sun W., Burdick J.A. (2017). A generalizable strategy for the 3D bioprinting of hydrogels from nonviscous photo-crosslinkable inks. Adv. Mater..

[bib21] Sakamoto H., Kumazawa Y., Motoki M. (1994). Strength of protein gels prepared with microbial transglutaminase as related to reaction conditions. J. Food Sci..

[bib22] Saunders R.E., Gough J.E., Derby B. (2008). Delivery of human fibroblast cells by piezoelectric drop-on-demand inkjet printing. Biomaterials.

[bib23] Shang L., Yu Y., Liu Y., Chen Z., Kong T., Zhao Y. (2019). Spinning and applications of bioinspired fiber systems. ACS Nano.

[bib24] Da Silva M.A., Bode F., Drake A.F., Goldoni S., Stevens M.M., Dreiss C.A. (2014). Enzymatically cross-linked gelatin/chitosan hydrogels: tuning gel properties and cellular response. Macromol. Biosci..

[bib25] Woodfield T.B., Malda J., De Wijn J., Peters F., Riesle J., van Blitterswijk C.A. (2004). Design of porous scaffolds for cartilage tissue engineering using a three-dimensional fiber-deposition technique. Biomaterials.

[bib26] Yang Y., Song X., Li X., Chen Z., Zhou C., Zhou Q., Chen Y. (2018). Recent progress in biomimetic additive manufacturing technology: from materials to functional structures. Adv. Mater..

[bib27] Yi H.G., Jeong Y.H., Kim Y., Choi Y.J., Moon H.E., Park S.H., Kang K.S., Bae M., Jang J., Youn H. (2019). A bioprinted human-glioblastoma-on-a-chip for the identification of patient-specific responses to chemoradiotherapy. Nat. Biomed. Eng..

[bib28] Yu Y., Shang L., Guo J., Wang J., Zhao Y. (2018). Design of capillary microfluidics for spinning cell-laden microfibers. Nat. Protoc..

[bib29] Yu Y., Chen G., Guo J., Liu Y., Ren J., Kong T., Zhao Y. (2018). Vitamin metal–organic framework-laden microfibers from microfluidics for wound healing. Mater. Horiz..

[bib30] Zeugolis D.I., Khew S.T., Yew E.S., Ekaputra A.K., Tong Y.W., Yung L.Y.L., Hutmacher D.W., Sheppard C., Raghunath M. (2008). Electro-spinning of pure collagen nano-fibres–just an expensive way to make gelatin?. Biomaterials.

[bib31] Zhao S., Riaud A., Luo G., Jin Y., Cheng Y. (2015). Simulation of liquid mixing inside micro-droplets by a lattice Boltzmann method. Chem. Eng. Sci..

