# Supplementary material for: Cascade Pumping Overcomes Hydraulic Resistance and Moderates Shear Conditions for Slow Gelatin Fiber Shaping in Narrow Tubes
Source: iScience. 2020 Jun 2;23(6):101228. doi: 10.1016/j.isci.2020.101228 (PMC7298654; doi:10.1016/j.isci.2020.101228)
Supplement: Document S1. Transparent Methods, Figures S1–S10, and Table S1 [file mmc1.pdf]

iScience, Volume *23*

Supplemental Information

Cascade Pumping Overcomes Hydraulic Resistance and Moderates Shear Conditions for Slow Gelatin Fiber Shaping in Narrow Tubes

Yuanxiong Cao, Haoran Zhao, Zhiwei Hu, and Shaohua Ma

Supplemental Information

Supplemental figures

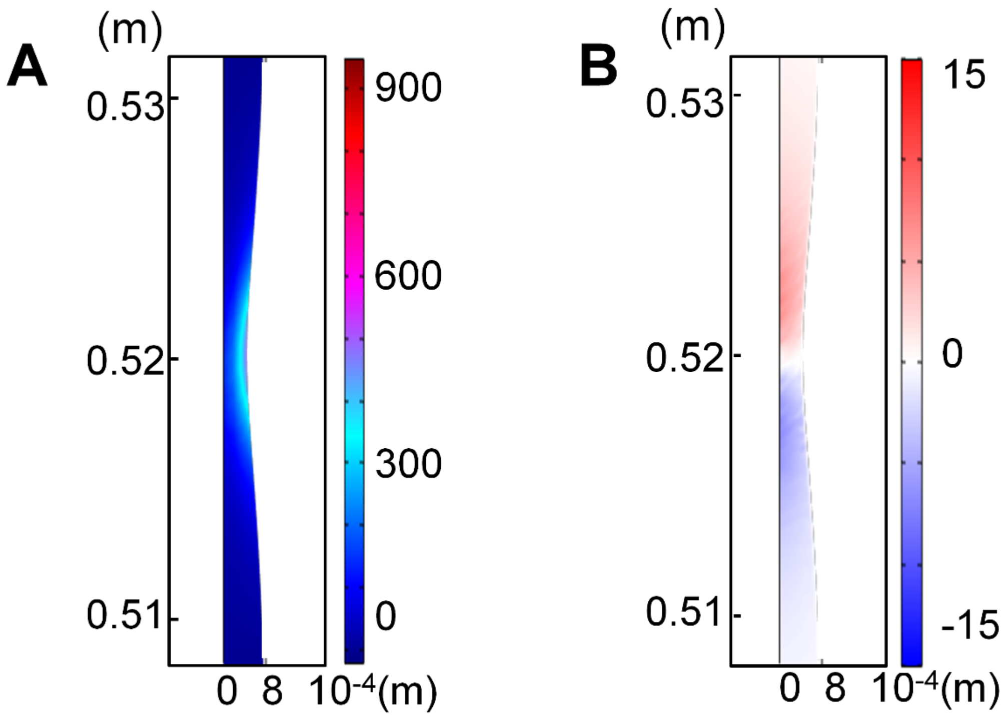

Figure S1. Shear rates of at the second pumping position of dual pumping: (A) in-plane shear rate, and (B) extensional strain. Related to Figure 2.

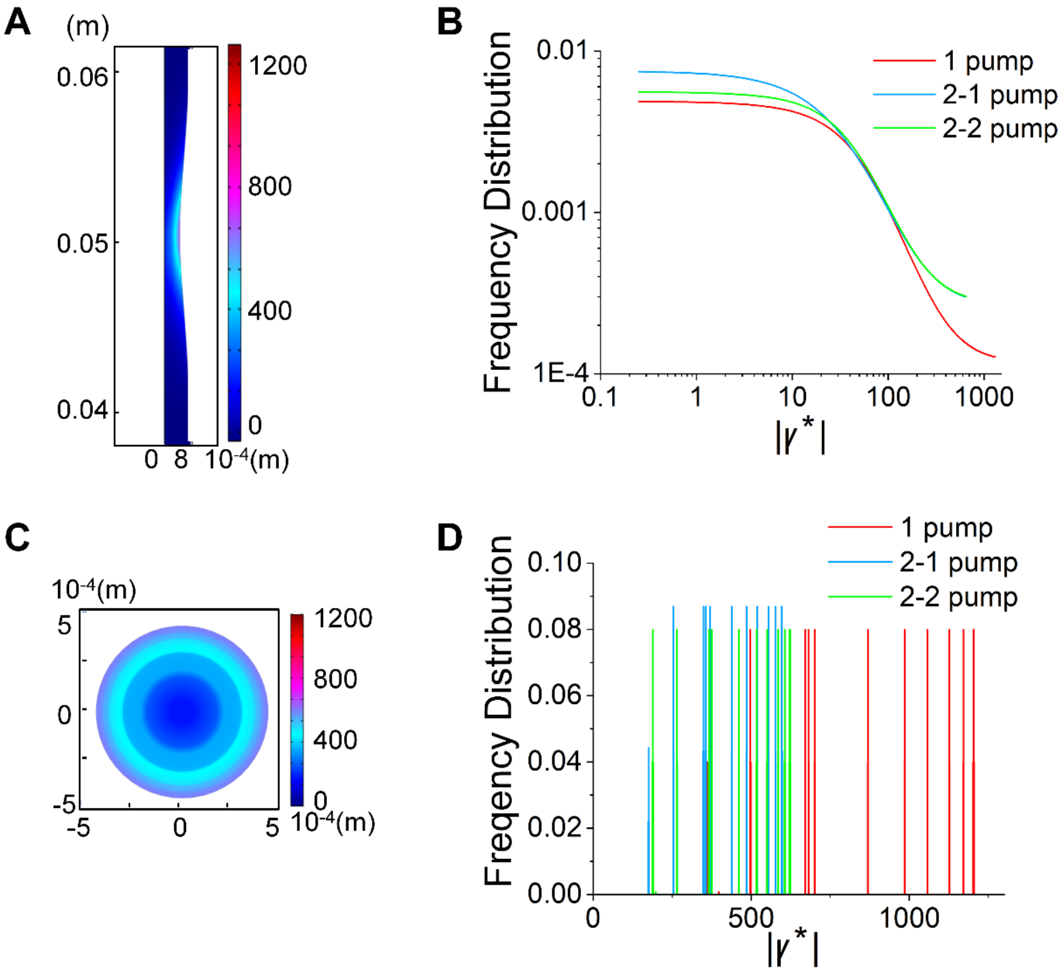

Figure S2. (A, C) Contours of shear rates and (B, D) their frequency distribution on (A) the *xy* and (C) the *yz* planes at the second pumping position of dual pumping. Merged frequency distribution of shear rate at *xy* (B) and *yz* (D) plane of single and dual pumps. Related to Figure 3.

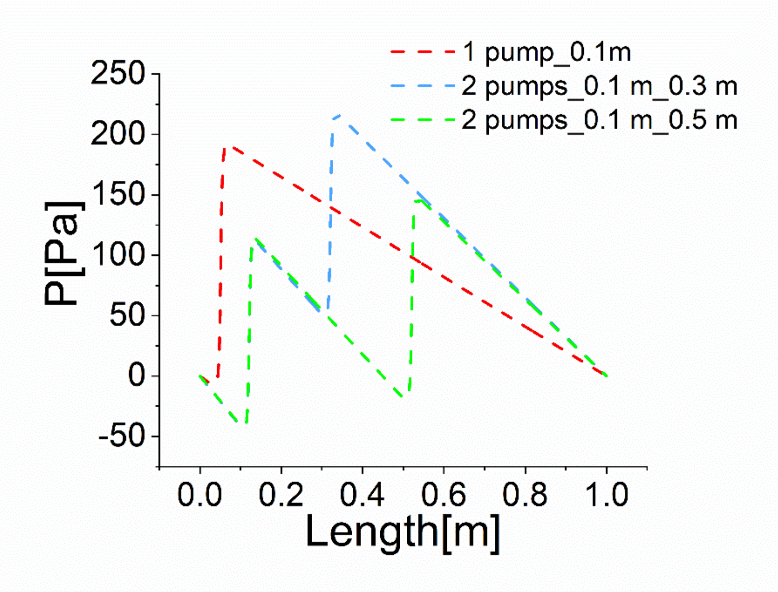

Figure S3. Pressure distribution along the fluid channel at *y* = r/2, along the tube axis, r is the radius of fluid for single and dual pumping, when applied the same amount of force on the driving fluid. Related to Figure 3.

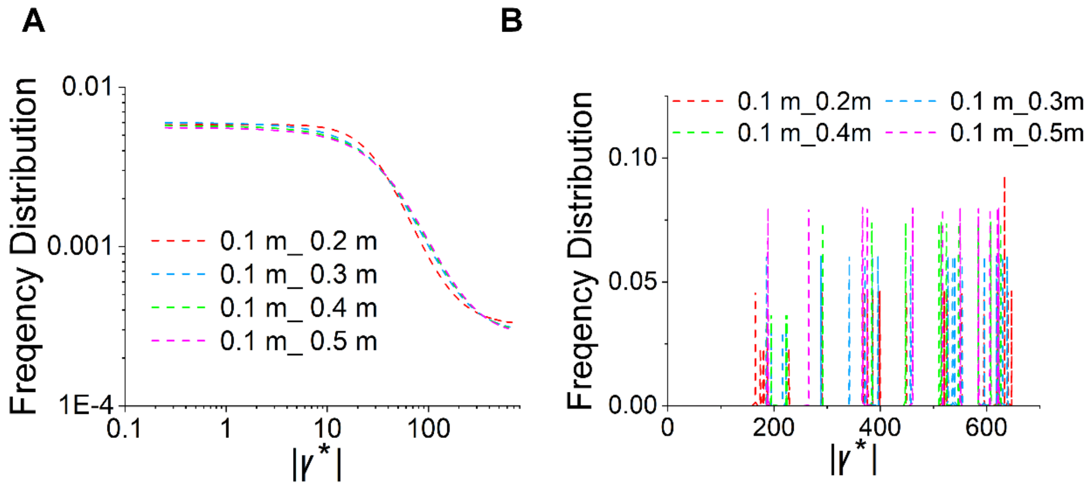

Figure S4. Frequency distribution of shear rates on the *xy* (A) and *yz* (B) planes at the secondary pumping position. Related to Figure 4.

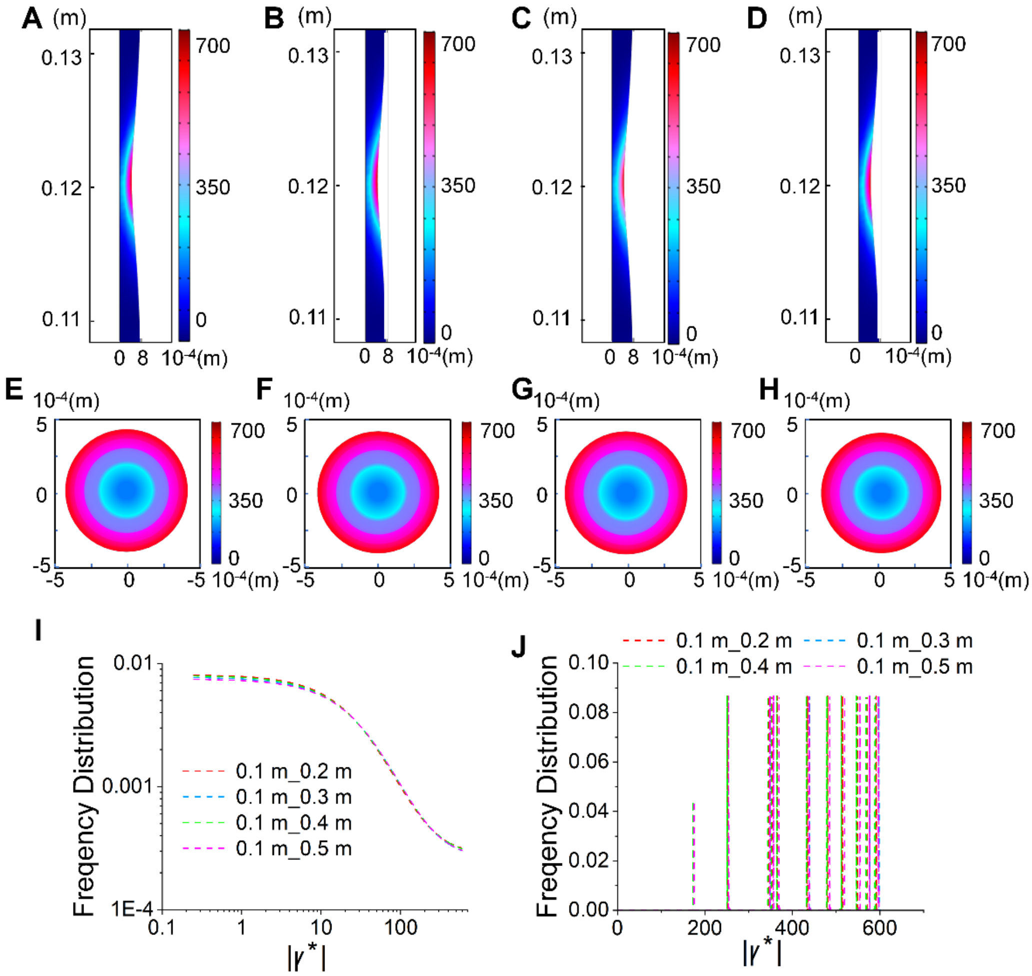

Figure S5. Contours of shear rates on the *xy* plane (A-D) and the *yz* plane (E-H) at the first pumping position. (I, J) Frequency distribution of the shear rates on the *xy* (I) and the *yz* (J) planes at the first pumping position. Related to Figure 4.

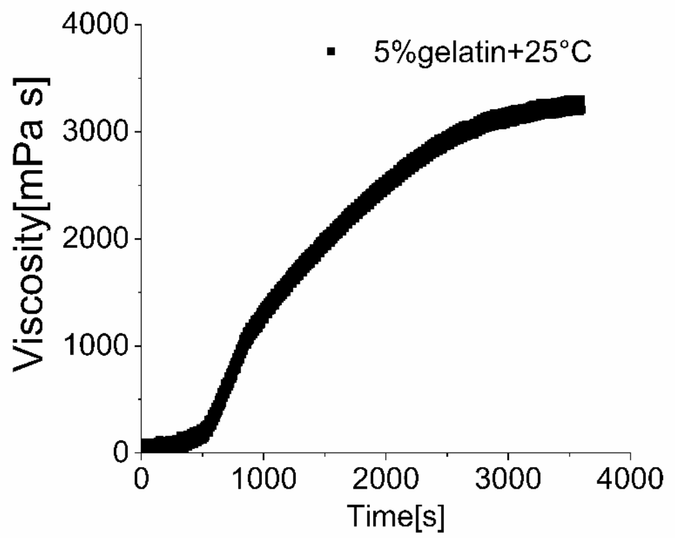

Figure S6. Time-dependent viscosity of 5% (w/v) gelatin solution at 25 ℃, the shear rate is 10 s^{-1}. Related to Figure 5.

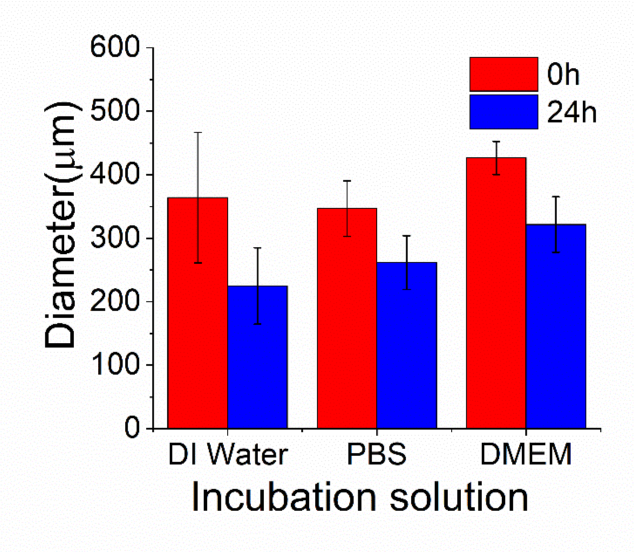

Figure S7. The diameters of gelatin/TG (25 °C) fibers before and after being incubated in DI water, PBS, and DMEM, respectively, for 24 h at 37 °C. Related to Figure 5.

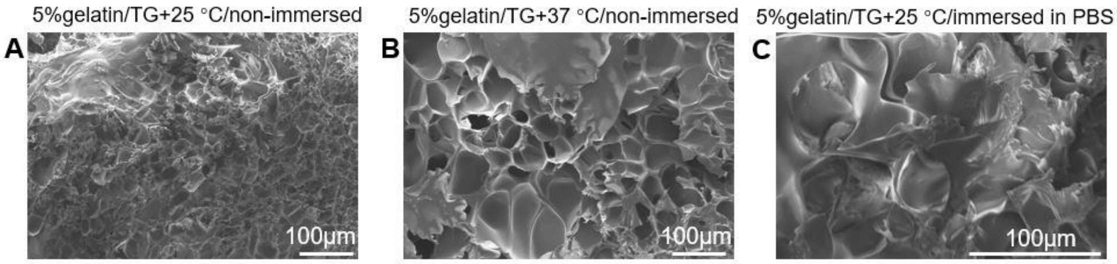

Figure S8. SEM characterization of freeze-dried 5% (w/v) gelatin/TG hydrogels. (A) A fresh (not immersed and incubated in any solution) 5% gelatin/TG fiber gelled at 25 °C. (B) A fresh 5% gelatin/TG fiber gelled at 37 °C (C) A 5% gelatin/TG fiber gelled at 25 °C and immersed in PBS for 24 h before imaging. Related to Figure 6.

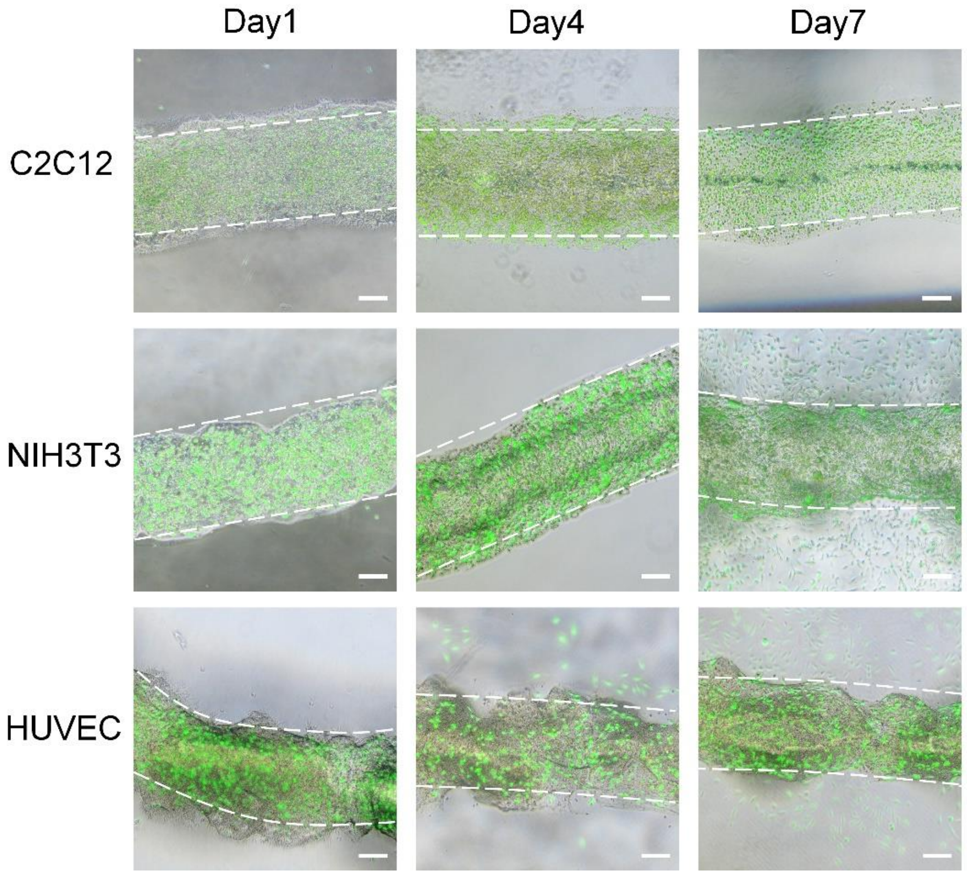

Figure S9. Merged fluorescence and bright-field microscopic images of cell-laden gelatin/TG fibers modulated by dual pumping formed at 25 °C. Fibers were laden with C2C12, NIH3T3, HUVEC cells and cultured for 1 day, 4 days and 7 days, respectively. Scale bars were 200 μm. Related to Figure 7.

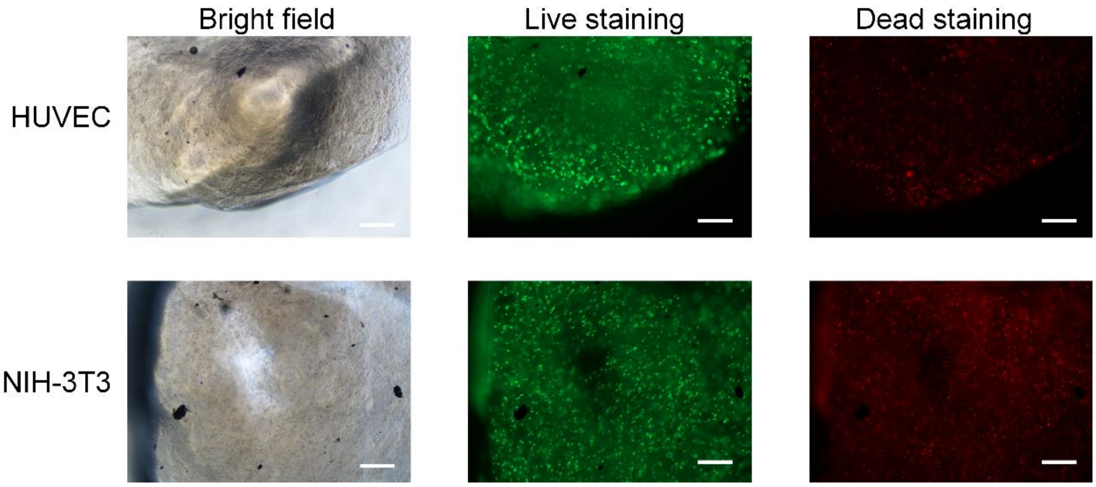

Figure S10. Fluorescence and bright-field microscopic images of cell-laden gelatin/TG fibers modulated by single pumping formed at 25 °C. Fibers were laden with HUVEC and NIH3T3 cells, they were stained just after being extruded by single pumping. Scale bars were 200 μm. Related to Figure 7.

Supplemental tables

Table S1. Parameters used in model simulation set up. Related to Figures 2,3,4.

**Parameter**	**Expression**	**Description**
tube_length	1 [m]	The tubing length
t_on	0.3[s]	Time when roll is engaged
t_off	1.2[s]	Time when roll is disengaged
dt	0.2[s]	Time to reach full force
z0	0.1[m]	First pump position
z1	0.5[m]	Second pump position
V_0	0.03[m/s]	Vertical velocity of roll
width	0.01[m]	Width of Gaussian force distribution
cycle	1.5[s]	Total time for a pump cycle
F_{max}	1.8e8[N/m^2]	Maxload

Transparent methods

Materials

Gelatin (Type A, 300 bloom from porcine skin) was purchased from Sigma–Aldrich (Wisconsin, USA). Transglutaminase was purchased from Dongsheng Biotech Co., Ltd (Taixing, China). Phosphate Buffered Saline (PBS) was purchased from Amresco (Shanghai, China). Dulbecco's Modified Eagle's Medium (DMEM), fetal bovine serum (FBS), penicillin-streptomycin, and trypsin were purchased from Basal media technologies Co., Ltd. (Shanghai, China). Endothelial cell growth medium (EGM) was purchased from Lonza (Germany). Calcein-AM/PI double stain kit was purchased from Yeasen Biotech Co., Ltd (Shanghai, China). Multi-channel peristaltic pump was manufactured by the Lead Fluid Technology Co., Ltd (Hebei, China). Water bath was purchased from Shangdao Instrument Manufacturing Co., Ltd (Shanghai, China).

The NIH-3T3 embryonic fibroblast cells and the C2C12 mouse muscle cells were purchased from China Center for Type Culture Collection. The HUVEC cells were extracted from three human umbilical cords. The cords were washed with PBS buffer twice. The trypsin (without EDTA) was injected through the vein and incubated at 37 ℃ for 15 min. The digested cells were cultured in EGM. The operation was repeated three times to extract enough cells.

Model simulation set up

The peristaltic pump model was set up axisymmetric used the fluid structure interaction model in comsol software. The 1 m long silicone tube has an inner radius of 0.8 mm and outer radius of 2.4 mm. A time and position dependent force is applied to the outer wall of tube, in the radius direction. The simplified force density of Gaussian distribution is applied at the pumping position, along the tube. The width of the Gaussian distribution is 1 cm, and it moves along the wall tube at a constant speed of 0.03 m/s. To represent the engagement of the roll, the force density, multiplied by a smoothed Heaviside function. The tube's deformation during a full cycle is 1.5 s.

The fluid flow is described by the incompressible Navier-Stokes equations (Eling et al., 2009):

$$\rho\frac{\partial\mathbf{u}}{\partial t}-\nabla\cdot\mu(\nabla\mathbf{u}+(\nabla\mathbf{u})^T)+\mu\mathbf{u}\cdot\nabla\mathbf{u}+\nabla p=0$$

$$\nabla\cdot\mathbf{u}=0$$

The equations are set up and solved in axial symmetry on a deformed mesh inside the tube.

The inlet and outlet pressure of tubing are both set as zero.

The parameters of setting are listed in Table S1.

Preparation of sample solutions

The gelatin fiber was synthesized by mixing gelatin with transglutaminase online, where the gradual enzymatic reaction occurred with prolonged incubation. The typical procedure was as follows: 6% (w/v) gelatin was dissolved in PBS buffer and then heated in an oven at 65 ℃ for 20 min to obtain a polymer solution. Next, the gelatin solution was kept in the water bath at 37℃ to stay as liquid. The gelatin solution was adjusted to pH = 7.0 by using 100 mM NaOH solution. Then, 60% (w/v) (TG) powder was dissolved in PBS buffer to obtain the saturated solution. The undissolved particles in suspension were removed by passing the solution through a 100 μm syringe filter.

Online extrusion

The gelatin and TG solution were loaded in two individual 50-mL centrifuge tubes as bio-ink cartridges, which were connected to two ports of a plastic T-shape connector via two flexible silicone tubes (1.6 mm inner diameter). A flexible tubing was connected to the third port in the connector and then fitted to a channel inside the pump casing. Afterward, the multi-channel peristatic pump rate was set at 5 r/min to withdraw gelatin and TG solutions. The two solutions were merged in the T-junction and thoroughly mixed when flowing along the long downstream tubing, where the enzymatic reaction occurred. The tube was then fitted to another channel in the pump casing, to consecutively withdraw and infuse the fluid. Then the gelatin solution was gelled and shaped into stable fibers when approaching the tubing outlet. The shaped fibers were then continuously extruded out of the outlet.

Rheology test

Gelatin and gelatin/TG solutions and hydrogels were evaluated by a rotational rheometer

(MCR302, Anton Paar, Austria). Measuring plate PP08 with a diameter of 8 mm was used in all measurements. The 5% (w/v) gelatin/TG (25 and 37 ℃) solutions were tested with viscosity-time scan in rotational mode at the shear rates of 5, 10, 15 s^{-1}, respectively. The 5% gelatin/TG (25 and 37 ℃) and gelatin (25 ℃) hydrogel were tested with viscosity-time scan in rotational mode at the shear rate of 10 s^{-1}. The shear stress and viscosity of 5% gelatin/TG (25 and 37 ℃) and gelatin (25 ℃) hydrogel were recorded by changing the shear rate from 0.1 to 10 s^{-1}. Storage moduli (G') and loss moduli (G'') were measured by changing the shear rate from 0.1 to 30 s^{-1}. Each measurement was repeated for three times.

Mechanical test

Mechanical properties of the gelatin hydrogels were performed using a universal testing machine (Mini Instron, USA) at room temperature. For compression tests, the gelatin/TG (25 and 37 ℃) and gelatin (25 ℃) were cured in the cultured dishes for 12 h. Each sample was cut into 8mm×10mm×4 mm and was placed between two compression plates and compressed at a displacement rate of 4 mm min^{-1}. For tensile tests, the gelatin/TG (25 and 37 ℃) and gelatin (25 ℃) hydrogel fibers with of 15 mm long and 1 mm wide were subjected to stretch at a strain rate of 10 mm min^{-1}. Each measurement was repeated for three times.

Scanning Electron Microscope (SEM)

The surface morphology exploring the structures and pore sizes of different gelatin/TG hydrogel constructs was observed using SEM (Hitachi SU8010). The 5% gelatin/TG fibers were immersed in DMEM medium for one week or kept in water bath at 25 and 37 ℃ for 24 h respectively. All samples were freeze-dried and putter-coated with platinum.

Cell encapsulation

The effects of crosslinking, mechanical stretching and compression on cell encapsulation were investigated. Human umbilical vein endothelial cells (HUVEC cells) were cultured in endothelial cell growth medium (EGM). NIH-3T3 mouse embryonic fibroblast cells and C2C12 mouse muscle cells were cultured in Dulbecco's modified eagle medium (DMEM) supplemented with

10% FBS and 1% penicillin-streptomycin. Afterward, the cells were suspended in gelatin solutions, at a density of 3×10^6 cells mL^{-1}. The cell-laden gelatin fibers were synthesized in similar manners as acellular fibers described before. The cell-laden fibers were cultured in the corresponding media for each cell types, in a humidified incubator at 37 °C with 5% CO_2 atmosphere. The medium was refreshed every other day.

Cell viability and imaging

Cell viability of HUVEC, C2C12 and NIH-3T3 in the cell-laden gelatin fibers was monitored by using the live/dead staining method. The double staining kit, Calcein AM (the live staining dye) and Propidium Iodide (PI, the dead staining dye), was used to stain live and dead cells in the fibers on day 1, day 4, and day 7 after fiber synthesis. The cell-laden fibers were washed three times with 1×PBS buffer. After that, they were immersed in 1mL PBS buffers supplemented with 1 µL 2 mM Calcein AM and 3 µL 1.5 mM PI for 1 h and then were washed three times with 1×PBS buffer before imaging under a fluorescent microscope (Nikon Elcipse Ts2r). The cell viability rate was quantified by counting the number of live (green) and dead (red) cells in the images.

Data processing

The rheology, mechanical tests, and frequency distribution data were processed using Origin software. The tensile tests data was smoothed to eliminate the noises. The frequency distribution curves of shear rate on *xy* plane were fitted using Lorentz function.

Supplemental References

Eling, C., Fouxon, I., and Oz, Y. (2009). The incompressible Navier–Stokes equations from black hole membrane dynamics. Phys. Lett. B. *680*, 496-499.
